# Real-Time Blink Detection as an Indicator of Computer Vision Syndrome in Real-Life Settings: An Exploratory Study

**DOI:** 10.3390/ijerph20054569

**Published:** 2023-03-04

**Authors:** Inês Lapa, Simão Ferreira, Catarina Mateus, Nuno Rocha, Matilde A. Rodrigues

**Affiliations:** Center for Translational Health and Medical Biotechnology Research, School of Health of Polytechnic Institute of Porto, 4200-465 Porto, Portugal

**Keywords:** computer vision syndrome, eye blink, blinking rate, eye-blink detection

## Abstract

With the increase in the number of people using digital devices, complaints about eye and vision problems have been increasing, making the problem of computer vision syndrome (CVS) more serious. Accompanying the increase in CVS in occupational settings, new and unobstructive solutions to assess the risk of this syndrome are of paramount importance. This study aims, through an exploratory approach, to determine if blinking data, collected using a computer webcam, can be used as a reliable indicator for predicting CVS on a real-time basis, considering real-life settings. A total of 13 students participated in the data collection. A software that collected and recorded users’ physiological data through the computer’s camera was installed on the participants’ computers. The CVS-Q was applied to determine the subjects with CVS and its severity. The results showed a decrease in the blinking rate to about 9 to 17 per minute, and for each additional blink the CVS score lowered by 1.26. These data suggest that the decrease in blinking rate was directly associated with CVS. These results are important for allowing the development of a CVS real-time detection algorithm and a related recommendation system that provides interventions to promote health, well-being, and improved performance.

## 1. Introduction

In occupational settings, computers are a crucial asset, as they facilitate the access to information, simplify communication, and improve performance [1,2]. However, it is important to realize that the workplace is no longer limited to an office and can be established anywhere, anytime [3]. The new working arrangements and the wide use of computers by knowledge workers have been increasing the time spent in front of the screen performing intensive computer tasks [4]. Additionally, it is important to recognize that these tasks are frequently related to unfavorable ergonomic conditions and poor viewing habits [4,5]. This has been related to musculoskeletal disorders and to a set of adverse visual and ocular effects [4].

In 1997, the American Optometric Association (AOA) stated that individuals who work long hours at a computer without any breaks report a higher level of complaints and symptoms, including stress, musculoskeletal problems, and ocular discomfort [6]. Despite the relevance of other symptoms, eye and vision disorders related to computer work have been emphasized. In fact, due to the increase in users requesting eye examinations related to the symptoms that they experience when working on the computer, the AOA defined computer vision syndrome (CVS) as a clinical entity [6]. Currently, this problem is even more prominent due to the amount of time spent by people using digital screens. Recent studies founded that 60–70% of office workers reported symptoms related to CVS [7,8].

CVS is described as a group of eye and vision-related problems that are experienced by subjects and that result from a prolonged use of a computer or other digital devices [4,9]. It has been related to a decrease in job satisfaction, poor visual function and physical well-being, increased stress levels, and reduced quality of life [10]. Therefore, CVS can significantly impair productivity in the workplace and increase absenteeism [11,12].

CVS can be assessed through a comprehensive eye examination, which should include visual acuity measurements, refraction testing, and a test to determine how the eyes focus, move, and work together [13]. Validated scales to determine CVS or dry eye have been proposed in the literature, such as the Computer Vision Syndrome Questionnaire (CVS-Q) [14] and the Dry Eye Questionnaire (DEQ) [15]. These scales usually address the eye and vision symptoms ranked by subjects. Due to the recent technological breakthroughs, new systems have been developed with the intention of diagnosing CVS, focusing on risk factor detection.

Several risk factors have been related to CVS; however, the relevance of maintained near vision without appropriate visual rest and for long periods of time, as well as limited blinking, have been noted in the literature. Several studies have found that frequent computer use is associated with an increase in incomplete blinks and a decrease in blink rate. This is likely due to the extended periods of visual attention required during computer work, which can lead to eye strain and fatigue (see, e.g., Blehm et al. [16]; Garg et al. [17]; Jennifer and Sharmila [8]; Kazuo Tsubota et al. [18]; Wimalasundera [3]). This decrease is thought to lead to the development of CVS, once it contributes to dry eyes [1]. 

While there are several instruments and exams available to detect computer vision syndrome (CVS) and measures that can help prevent it, there remains a gap in the availability of unobtrusive smart systems that can detect risk factors and assess the risk of CVS in real time during day-to-day computer use. It is important that these systems be prepared to be used during day-to-day activities, considering the real-life conditions that determine the interface between subjects and the computer. To address this gap, we aimed to investigate whether blinking data collected from a computer webcam in real-life settings can be used as a reliable indicator for predicting CVS. The goal was to obtain inputs to develop a real-time CVS detection algorithm and a related recommendation system that provides interventions to promote health and well-being and improve performance. This study will help bridge the gap between existing CVS detection tools and real-life computer usage conditions, providing a more comprehensive understanding of the condition and the means to effectively address it.

### 1.1. Computer Vision Syndrome (CVS)

CVS is an ocular condition characterized by a set of eye-related symptoms that appear to increase during operating or looking at a digital screen [9]. Table 1 summarizes the symptoms related to CVS, according to Blehm et al. [16]. When a person is screened as having one or more of these symptoms, they are commonly diagnosed with CVS [3,14]. The prevalence of CVS among workers who use digital screens is high. Chu et al. [19] identified CVS-related symptoms in more than 70% of computer users. Similar results were obtained by Agarwal et al. [20].

Vision and eye problems experienced while working on a computer or other screen device are often temporary and may improve after the activity has ended [21,22]. However, some workers experience symptoms, such as blurred distance vision, even after work [23].

### 1.2. Blinking 

Blinking is a temporary closure of both eyes involving movements of the upper and lower eyelids. This motion is important for ensuring the maintenance of the ocular surface integrity. Blinking contributes to the maintenance of ocular surface moisture and favors the drainage of tears [24]. On average, a person blinks 15–20 times per minute; however, studies show that this rate is significantly lower when working at a computer, decreasing to 4–6 times per minute [1,3,8,16,17,25]. This is a relevant cause of CVS, since the decrease in blink rate increases the exposure of the ocular surface area, which leads to a poor tear film quality and temporarily stresses the cornea, causing eye dryness [3,5,16,17,26].

Computer use has been associated with an increase in incomplete blinks and a reduced blink rate when compared with viewing hard-copy materials [27]. In a study of 104 office workers, the blink rate was reduced from 22 times per minute in a relaxed state to 10 times per minute when reading a book and 7 times per minute in the visual display terminal (VDT) [28]. That happens because the eyes do not have a problem focusing on printed materials, which are distinguished by dense black characters with well-defined edges. However, characters on a screen do not have this contrast or well-defined edges, which makes it difficult for the eyes to maintain focus and fixation on these images. It has also been reported that the rate decreases as contrast and letters size are diminished, as well as with increased demand of the task and spacing between characters and lines [5,16,27]. This problem is enhanced due to a long-lasting viewing period causing strain in the optical system and contributing to eye dryness [1]. 

### 1.3. Systems That Determine Blink

There is a significant body of literature describing various research studies that utilize eye movements to develop eye blink detection systems. Table 2 summarizes some of the existing systems and the methods adopted to determine blinking.

In the last 10 years, different methods have been developed to determine rapid blink movements. In fact, since blink is so rapid, special techniques are required to characterize it. A non-exhaustive list of these methods ranges from the use of the position of the eyelids, variation of pixel pigmentation, and patterns of eye aspect ratio values. Previous studies emphasized the use of cameras to this end. In fact, real-time systems can be adopted using a simple computer webcam. This carries a high potential to be used also in the field of occupational safety and health as, for example, to predict CVS. However, none of the studies have associated blinking with CVS, despite this relevance being emphasized by Worah et al. [29].

**Table 2:** Summary of studies that address systems to determine blink.

Authors	Title	Objective	Technology Used	Methods of Blinking Detection
Bernard et al. [30]	Eyelid contour detection and tracking for startle research-related eye-blink measurements from high-speed video records	Present a semi-automatic model-based eyelid contour detection and tracking algorithm for the analysis of high-speed video recordings from an eye tracker.	Detection of blink through high-speed video recordings	Use of positions of the eyelids for the measurement of eye blinks. Use of several landmarks to update eye shape. The distance is estimated between lower and upper eyelids to represent the blink
Gisler et al. [31]	Automated detection and quantification of circadian eye blinks using a contact lens sensor	Detect and quantify eye blinks during 24-hour intraocular pressure (IOP) monitoring with a contact lens sensor (CLS).	Contact lens sensor	Detect and quantify eye blinks for 24 h. Intraocular pressure monitoring with a contact lens sensor.
Jennifer & Sharmila [8]	Edge based eye-blink detection for computer vision syndrome	Develop a prototype using blink as a solution to prevent CVS.	Computer or laptop webcam	The frames are processed for detecting the eye status based on the edges by using direct pixel count, gradient, Canny edge and Laplacian of Gaussian (LoG). No relation with CVS was performed.
Le et al. [32]	Eye blink detection for smart glasses	Describe an approach to eye-blink detection that suits low-power platform well, such as smart glasses	Smart glasses equipped with a low-power camera	Gradient boosting (GB) algorithm to learn the eye-blink patterns based on the closing-eye detection results.
Noman & Ahad [33]	Mobile-based eye-blink detection performance analysis on android Platform	Develop a real-time gaze tracking and eye-blink detection system that operates on a simple Android mobile phone having a frontal camera	Android mobile phone with a frontal camera	Blink detection based on the time difference between two open-eye states, where the open eyelid is taken as a template for detecting eye blink.
Galab et al. [34]	Adaptive real time eye-blink detection system.	Proposes a webcam system for detecting eye blinks	Laptop Webcam	Blink is determined by the difference between the number of black pixels in the bottom part of the eye object to the number of black pixels in the above part (eye is closed when the difference higher than zero)
Mohammed and Anwer [35]	Efficient eye blink detection method for disabled helping domain	Proposes a real-time method based on some video and image processing algorithms for eye blink detection.	Android mobile phone with a frontal camera	Eye-tracking algorithm that considers the position of the detected face. Blink detection based on eyelid states (closed or open). This approach has explored a smoothing filter to enhance detection rate.
Soukupova and Cech [36]	Real-time eye- blink detection using facial landmarks	Develop algorithm that detects eye blinks in a video sequence	Standard camera	Detect eye blinks as a pattern of eye aspect ratio values that characterize the eye opening in each frame.
Worah et al. [29]	Monitor eye care system using blink detection, a convolutional neural approach	Develop a system that detects when the blink rate falls below the normal threshold to prevent CVS	Webcams mounted on the monitor screens, inbuilt laptop webcams and front camera of mobile phones	Algorithm based on convolutional neural network to detect eye states and to predict blinks. Eyeblink detection is made up of four phases: (1) image capture, (2) face detection, (3) eye localization, and (4) blink detection
Dewi et al. [37]	Adjusting eye aspect ratio for strong Eye-blink detection based on facial landmarks	Proposed a real-time method that detects eye blinks in a video series	Camera installed on car dashboard	Estimates the facial landmark positions and extracts the vertical distance between the eyelids using the facial landmark positions relative to the landmarks to determine the degree of eye-opening and closing.

## 2. Materials and Methods

### 2.1. Study Design

This study was a longitudinal exploratory study for the detection and monitoring of eye variables made through facial recognition software installed in the participants’ computers, which further collected users’ physiological data through the computers’ cameras without storing any kind of image or video, thereby safeguarding personal information. This is very important to address, since this solution can analyze video data without storing or recording any image feed, allowing for real-time analysis while preserving data governance. The metric used in this preliminary study was the blinking rate during a working day. Additionally, this study matched the data provided by the system against a CVS scale. Thus, the data was used to contribute to the validation of the entire system.

To guarantee anonymity, data collected from the subjects were encrypted and stored only on their personal computers. After data collection, each subject (data holders) shared the data with the research team. The investigation complies with the principles recommended by the Declaration of Helsinki, and it was previously approved by the Ethics Committee of the School of Health, Polytechnic Institute of Porto. At the beginning of the study, the objectives and procedures involved were explained to all participants. Additionally, each participant completed a digital consent form, including their personal signature.

### 2.2. Sample

Thirteen students were included in the study. Among the subjects, there were 10 females and 3 males with ages ranging from 21 to 25 years. As inclusion criteria for this study, subjects had to do daily computer work (at least four hours a day), be 25 years old or younger, and be enrolled in a scientific writing task for the whole period of data collection. It was found that the group in this study spent an average of 20 h on the computer per week. Unfortunately, one participant had to drop out due to alleged computer issues, and we excluded another participant due to poor data quality, leaving the final sample included in the analysis with eleven participants.

The participants were instructed to follow a code of conduct that involved starting the webcam monitoring software whenever they were working on a scientific writing task for a period longer than 1 hour. They were also instructed to be in front of the screen for at least 4 hours every day and to attach their webcam so that it was facing them. It was also important for participants to ensure that they were in a lit environment while using the webcam monitoring software to maintain the integrity of the study and ensure that the data collected was accurate and reliable. Before starting the protocol for data collection, participants were instructed on the software’s installation, questionnaire completion, and data governance. They also had preliminary training about ergonomic requirements for computer tasks (workstation design, visualization distance, illuminance, and eye resting).

### 2.3. Determination of Eye Blink

In this research, we used the “HealthyRoad” software developed within the “Mad@Work” project to determine eye blink. “HealthyRoad” is a webcam-based facial biometric software that collects physiological data, including heart rate, blinking, and emotions. This application does not collect any type of image or video and only saves numerical data. Data are saved on the user’s computer and can only be shared by the data holder, i.e., the participant. This is one of the few solutions with this potential available in the market.

Blinking rate was the main variable used. For this, we had to compute the blinking rate using the number of blinks collected by the system, the frame rate that every participant’s computer was assuming, and the acquisition rate. Every blink was determined according to the percentage of eye opening. A single blink corresponded to a percentage of 0, being that both eyes needed to be closed at a certain point to address a single blink. The software has been through a thorough impact assessment and the data collected will not be used beyond the scope of scientific publications and aggregated dissemination.

### 2.4. Determination of CVS

All the participants filled out a questionnaire to assess CVS. The Computer Vision Syndrome Questionnaire (CVS-Q) is a scale developed by Segui et al. (2015) [14] and validated for Portuguese language by Rodrigues e Mateus (2020). The CVS-Q assesses the frequency and intensity of 16 symptoms related to CVS.

The symptoms included in the questionnaire were burning, itching, feeling of a foreign body, tearing, excessive blinking, eye redness, eye pain, heavy eyelids, dryness, blurred vision, double vision, increased sensitivity to light, difficulty focusing for near vision, halos around objects, feeling that vision is deteriorating, and headache. To measure how frequently these symptoms occur used a rating scale was used, with the following categories: never (this symptom doesn’t occur), occasionally (sporadic episodes or once a week), and often (two or three times a week)/very often or always (almost every day). The intensity or strength of the symptom was measured on a 2-point scale: moderate and intense. Symptoms that were reported as “never”, in terms of frequency, were not evaluated for their intensity. The severity index (frequency × intensity) of visual symptoms was determined according to the measurement scale validated by Segui et al. (2015) [14]. The score was obtained by applying the following equation. If the score obtained was ≥6, they were classified as a CVS case category.

$$Score=\overset{16}{\underset{i=1}{\sum }}{\left(frequency\;of\;symptom\;occurrence\right)}_{i\;}\times {\left(intensity\;of\;symptom\right)}_{i}$$

### 2.5. Data Collection and Blinking Rate

The data holders shared all the data collected with the researcher team. The “HealthyRoad” software transforms all physiological data into numerical outputs and saves it in csv files. These files were statistically analyzed in RStudio version 2022.02.2 Build 485, using primarily *Dplyr* to manipulate the data. Every computer addressed a different data acquisition frame rate, according to the computer’s processor and RAM capabilities. The acquisition frame rate ranged from four frames per second (fps) to 30fps for each csv file. The frame rate was computed based on the acquisition period and frames acquired.

To define quality in our data, we started by eliminating every file that contained data collection below 2 minutes or 500kb of data. We considered data files that had at least 1 hour of screen time at each instance. Considering that every participant can have different desktop setups, various numbers of monitors, or even perform different tasks in front of the computer, we always addressed a threshold of 75% face detection in every file, maintaining this threshold for being in front of the screen. We excluded 23 data collection files from the analysis, having in mind the thresholds described above.

### 2.6. Statistical Analysis

Regarding descriptive statistics and taking into account the small sample size, absolute and relative frequencies were computed for categorical variables, while mean and standard deviation were reported for continuous variables, as shown in Table 3. For inferential analysis, correlations were used to test the association between CVS, daily blinking rate, and mean blinking rate for each participant, as well as age, as shown in Table 4. To compare groups regarding CVS, independent samples t-test was applied (males vs. females and using glasses). According to the guidelines of Gignac and Szodorai [38], the magnitude of zero-order correlations was classified as small (≥0.10), moderate (≥0.20), and large (≥0.30) [38]. Finally, in order to estimate test statistics and confidence intervals for the independent samples t-tests (Table 4) and zero-order correlations (Table 5), as the normality assumption was not met for a number of analyses, bootstrapping was used (bias-corrected accelerated, 1000 samples). Having in mind the previous inferential tests, a regression model was prepared, including all variables significantly and marginally associated with CVS. 

The following assumptions were tested before implementing the regression model: normally distributed residuals (examination of standardized residual distribution and identification of influential cases), homoscedasticity (Koenker test and visual examination of the scatter plot of absolute standardized residuals versus standardized predicted values), collinearity (correlation matrix between independent variables), multicollinearity (tolerance and variance inflation factor). 

When necessary, the regression model was adjusted following assumption testing.

All analyses were carried out using IBM SPSS (version 26). To control for multiple analyses, significance levels were set at 0.05.

## 3. Results

Sample characteristics are described in Table 3. Despite the attempt to include the same number of subjects of each gender, the majority were female (81.8%). Still, it was observed, in this study, that gender was not associated with differences in CVS (*p* = 0.342, Mdiff 95% CI [14.00, 22.18]), as both males and females displayed quite similar scores (M = 22.00, SD = 2.8 vs. M = 17.22, SD = 6.38, respectively). However, CVS severity results were negatively and highly correlated with age (r = −0.639, *p* = 0.034, 95% CI [−0.957, −0.148]), showing as the higher the age of the subjects, the lower the CVS severity.

Obtained results also showed that CVS was negatively and highly correlated with average of blinking rate (r = −0.781, *p* = 0.005, 95% CI [−0.927, −0.248]), i.e., the lower the average blinking rate, the higher the CVS.

### Regression Model

A regression model was prepared including, as predictors, all the variables significantly related to CVS from the previous analyses (average blinking rate and age) as well as gender, since it is a referenced variable that has an impact on CSV. Table 4 and Table 5 present the findings.

Several assumptions were tested before proceeding with the final model. Regarding the normally distributed residuals assumption, standardized residuals displayed an approximately normal distribution with no evidence of collinearity or multicollinearity. Finally, the scatter plot with absolute standardized residuals by standardized predicted values did not suggest any degree of heteroscedasticity. Thus, the regression model was run using the wild bootstrap, which does not assume homoscedasticity [39,40]. 

Results from the final regression model are presented in Table 6. A significant regression equation was found (F (3,7) = 7.685, *p* = 0.013, with an R2 of 0.876). Average blinking rate was the only significant predictor of CVS (B = −1,26, *p* = 0.019, 95% CI [−2.249, −0.275]), meaning that, on average, for each added blink the CVS score decreased by 1.26. Age and gender were not significant predictors of CVS (B = 0,36, *p* = 0,84, 95% CI [−3.742, 4.462], and B = 6,16, *p* = 0.071, 95% CI [−0.702, 13.023], respectively).

## 4. Discussion

The current study aimed to determine whether a smart system can collect eye variables, particularly blinking rate, and predict CVS, in order to develop a real-time detection algorithm and a linked recommendation system that improves performance and provides interventions that promote health and well-being. 

There have been a small number of published studies about blink rate determination. The existing ones showed that the average blinking rate is about 15 to 20 times per minute, decreasing to 4 to 6 times per minute during computer usage [28,41]. A reduced number of blinks per minute when using the computer contributes to an increase in symptoms associated with CVS, as there is no constant renewal of the tear film [8]. Results on blinking rate can vary during computer usage. Eye blinking rate increases during speaking [42], visual fatigue [43], and sleep deprivation [44]. In contrast, eye blinking rate decreases during computer work [1,3,16,17,25] and demanding visual tasks [44]. In this study, data was collected during computer usage requiring high demand in visual tasks and high concentration, ensuring the relevance of blink rate for CVS development.

For analysis of the CVS, several subjective methods can be used based on the perception of symptoms, and then evaluated regarding their frequency and intensity, such as questionnaires [14]. In this study, the CVS-Q scale was applied and the score determined. Individuals who obtained a score of 6 or higher were categorized as having symptoms of CVS. Our results show that, on average, for each extra blink, the CVS score dropped by 1195. This is a clear indication that computer visual syndrome is caused by a lower blinking rate, which suggests that it is possible to use the blinking rate as a diagnostic tool to identify the syndrome. This can be highly valuable for providing real-time guidance to the subjects, helping them to reduce the severity of the syndrome with subtle interventions, for example, instructing the subject to increase their blink rate when using the computer or taking a short break.

In this study, other variables significantly associated with CVS were used. Gender was integrated into the model as a covariable, being a personally relevant variable, since there are studies that indicate a significant blinking rate difference between males and females, being that women present a lower frequency [45]. However, the prevalence of symptoms associated with CVS has been reported to be higher in females (see, Portello et al., 2012 [46], Rahman and Sanip [47]; Ranasinghe et al. [48], Taino et al. [49], Tauste et al. [50], Uchino et al. [26], Wiholm et al. [51]). This fact may be related to the high prevalence of dry eye among female subjects.

We also included age in our model, despite other studies stating that elderly individuals have shorter duration of blinks and longer intervals between blinks [45]. In our study, we found no statistical evidence of an age difference, mainly because of the low standard deviation.

In this study, it was possible to conclude a step forward in relation to previous studies, since the relation of the blink and the CVS using a smart system was established. Worah et al. [36] developed a system to detect low blink rate in order to prevent CVS. The authors only focused on blink detection to determine whether the blink rate was low, rather than obtaining the exact blink rate. However, no relation with the CVS was established. Also, Jennifer and Sharmila [8] proposed a system prototype to determine blinking rate as a solution to prevent CVS. However, there was still no relation with CVS symptoms. 

## 5. Limitations

The present research and its results must be addressed carefully, as there are several limitations. We must endorse that this study has a longitudinal exploratory design in real-life settings. From our knowledge, this is the first attempt to start designing an algorithm that could have the ability to predict CVS based on real-time blinking rate. Although the results are very promising and point future research in the right direction, sample size and gender class imbalance were two of the biggest limitations. It is important to notice that despite the high granularity of data, subjects were not in front of the computer for a restricted number of hours or a specific time schedule. We would also like to mention that some machines would go as low as four fps, which can result in a limitation for the software and its operability in older machines. 

These results are still preliminary. Further research is needed to validate the software and its usage. A higher sample size and a more restricted schedule is important for addressing data comparability.

## 6. Conclusions and Future Investigations

The present research aimed to determine whether a smart system can predict CVS through collecting eye variables in real-life settings. A significant prediction was found, pointing towards average blinking rate being capable of predicting CVS. This presents a powerful and meaningful change in the CVS detection field. We believe that this study can open the door to new developments in the understanding of CVS, its prediction, and prevention using intelligent systems. 

Future research is needed to continue to elevate CVS detection and prediction models. What we want to achieve is to understand how the syndrome is developed in different occupational settings. We are developing this concept with larger samples and with knowledge workers. It would be pertinent for the system to provide interventions that promote health and well-being and improve performance. Other eye-related variables, such as eye gaze and pupil diameter, should be addressed and inserted into the model to determine CVS. This would be particularly interesting since eye gaze has been used as an indicator of the viewing angle, denoting higher ocular exposure to the environment and poorer workplace designs. Likewise, pupil diameter has previously been used as an indicator of visual fatigue, given that demanding tasks cause an increase in pupil size.

## Figures and Tables

**Table 1:** Symptoms related to CVS (adapted from Blehm et al. [16]).

Category	Symptoms	Causes
Asthenopia	Eyestrain Tired eyes Sore eyes Dry eyes	Binocular vision Accommodation
Ocular surface-related	Irritation Watery eyes	
Visual problems	Blurred vision The slowness of focus change Double vision	Refractive error Accommodation Binocular vision
Extraocular (ergonomic problems)	Back pain Neck pain Shoulder pain	

**Table 3:** Sample characteristics (n = 11).

	Mean ± SD	Min—Max
**Age**	22.00 ± 1.00	21–25
	**Groups**	**N (%)**
**Gender**	Female	9 (81.8%)
	Male	2 (18.2%)
**Glasses**	Yes	4 (36.4%)
	No	7 (63.6%)

**Table 4:** Group Comparison (independent t-test).

Group Comparison						
	Yes Mean ± SD (n)	No Mean ± SD (n)	*t*	*p*	Mean Difference	95% Cis
**Glasses**	16.75± 6.90 (n = 4)	18.86 ± 6.01 (n = 7)	0.532	0.608	2.107	[−6.855, 11.070]
**Gender**	Male Mean ± SD (n)	Female Mean ± SD (n)	*t*	*p*	Mean Difference	95% Cis
	22.00 ± 2.82 (n = 2)	17.22 ± 6.38 (n = 9)	−1.004	0.342	−4.778	[−15.544, 5.988]

**Table 5:** Summary of Pearson’s correlations.

Measure	M	SD	2	3	4	5	6	7
1. CVS	18.09	6.09	−0.517	−0.870 **	−0.260	−0.357	−0.575	−0.639 *
2. Day1	12.79	5.26		0.323	−0.150	0.479	0.340	0.305
3. Day2	13.92	7.72			0.598	0.324 0.108	0.678 * 0.360 0.151	0.792 **
4. Day3	11.14	6.49						0.562
5. Day4	13.59	5.47						0.160
6. Day5	13.62	5.35						0.609 *
7. Age	22.00	1.00						−0.134

* *p* < 0.05; ** *p* < 0.001.

**Table 6:** Predictors of computer vision syndrome.

	B	SE B	B 95% Cis	β
Average Blinking	−1.262	−0.865	[−2.249, −0.275]	0.019 *
Age	0.360	0.060	[−3.742, 4.462]	0.842
Gender	6.160	0.409	[−0.702, 13.023]	0.071
R2			0.876	
F			7.685 *	

* *p* < 0.05.

## Data Availability

Not applicable.
